# A Case of Pulmonary Thromboembolism in a Child With Steroid-Resistant Nephrotic Syndrome

**DOI:** 10.7759/cureus.102260

**Published:** 2026-01-25

**Authors:** C. Annette Reyes, Ei Khin

**Affiliations:** 1 Pediatrics, Texas Tech University Health Sciences Center El Paso Paul L. Foster School of Medicine, El Paso, USA; 2 Pediatric Nephrology, Texas Tech University Health Sciences Center El Paso Paul L. Foster School of Medicine, El Paso, USA; 3 Pediatric Nephrology, El Paso Children's Hospital, El Paso, USA

**Keywords:** anticoagulants management, nephrotic syndrome, pulmonary embolism, steroid resistant nephrotic syndrome, thromboembolism

## Abstract

A seven-year-old girl with steroid-resistant nephrotic syndrome (SRNS) presented to the Emergency Department with chest pain, palpitations, dyspnea, and generalized edema. She had been receiving immunosuppressants but remained nephrotic. During evaluation, she was found to have an acute occlusive pulmonary thromboembolism (PTE), confirmed by CT pulmonary angiography. Anticoagulation was initiated promptly with heparin, followed by enoxaparin, a low-molecular-weight, long-acting heparin.

This case highlights the increased risk of thromboembolic events in children with SRNS due to their hypercoagulable state. It underscores the importance of vigilant SRNS management, awareness of life-threatening complications, such as pulmonary embolism (PE), timely imaging, and prompt treatment.

## Introduction

Thromboembolism (TE) is a notable but rare complication in children diagnosed with nephrotic syndrome (NS), with a significant risk in those with steroid-resistant nephrotic syndrome (SRNS). Pediatric NS has an estimated incidence of approximately 2 to 7 per 100,000 children globally, making it one of the most common pediatric kidney diseases [1,2]. NS is characterized by a triad of hypoalbuminemia, edema, and hyperlipidemia, primarily driven by nephrotic-range proteinuria, which is defined by a spot urine protein-to-creatinine ratio >2 by the Kidney Disease Improving Global Outcomes (KDIGO) guidelines [3]. Patients with SRNS, clinically characterized by persistent significant proteinuria despite corticosteroid treatment, exhibit a higher likelihood of encountering TE [1,2]. In a systematic review involving 14,290 children from 22 studies, the prevalence of TE in patients with NS was 3.5%. In two studies evaluating TE in relation to NS histology, the reported rates were 1.5% in steroid-sensitive nephrotic syndrome (SSNS) and 6.3% in SRNS [4].

The mechanism behind the increased risk of venous thromboembolism (VTE) in NS relates to a hypercoagulable state induced by significant proteinuria. This state arises from the urinary loss of antithrombin III, protein C, and protein S anticoagulants, along with compensatory increases in hepatic production of various procoagulant factors, such as fibrinogen and von Willebrand factor [2,5]. SRNS carries a higher risk of TE due to persistent heavy proteinuria and sustained hypoalbuminemia that maintains a long-standing hypercoagulable state.

Although the overall risk of thrombus formation is generally low at approximately 3%, it rises significantly in congenital NS or associated conditions like lupus nephritis; the incidence of TE can exceed 25% [1,2].

Key risk factors that amplify the likelihood of VTE in NS include significant proteinuria, immobility, volume depletion, central venous access, infection, and immunosuppressive therapy [1,2]. Children with NS may present with non-specific symptoms when facing pulmonary embolism (PE), complicating timely diagnosis and treatment. This clinical presentation is often subtle, necessitating heightened vigilance and a high index of suspicion among healthcare providers managing pediatric populations [6].

## Case presentation

A seven-year-old girl with a history of SRNS and hypertension for two months presented to the Emergency Department with chest pressure, palpitations, and dyspnea for one day. She denied fever, cough, syncope, headache, joint pain, rash, bruising, abdominal pain, or bleeding.

She had been hospitalized multiple times for persistent nephrotic range proteinuria associated with worsening edema and fever. The current presentation occurred one day after discharge from a six-day hospitalization during which she underwent a renal biopsy and received three doses of pulse methylprednisolone (30 mg/kg), a single dose of 25% albumin with furosemide, and continued tacrolimus and prednisone. Since her initial diagnosis, she had remained nephrotic with a 24-hour urine protein of 5.4 g/day. She did not have central venous catheter placement.

Her past medical history included prematurity (32 weeks’ gestation), birth weight 4 lb 8 oz, and twin delivery by elective cesarean section. She required a 26-day NICU stay for feeding difficulty and oxygen support. Additional diagnoses included atrial septal defect/patent foramen ovale (ASD/PFO) and mild intermittent asthma. Current medications were lisinopril 5 mg two times a day, tacrolimus 2 mg two times a day, prednisolone 30 mg every other day, famotidine, and albuterol.

Family history was notable for nephrotic syndrome in her twin sister and a history of childhood renal disease in a paternal uncle, which subsequently resolved. There was no known family history of thrombosis or bleeding disorders. She lived with her parents and three siblings. Immunizations were current, except for the influenza and COVID-19 vaccines for that season.

Upon arrival, the patient appeared anxious but remained alert, weighing 21.9 kg. She was tachycardic with a heart rate of 102 beats per minute and tachypneic with a respiratory rate of 22 breaths per minute, and signs of mild dehydration. Her blood pressure was elevated at 129/98 mmHg, and she was afebrile with a temperature of 36.6°C. Her oxygen saturation was 91% on room air. She demonstrated generalized edema, and breath sounds were diminished at both posterior lung bases without any adventitious sounds. Cardiac examination revealed no murmurs, and peripheral pulses were palpable. Her abdomen was distended with ascites, dull to percussion, and exhibited a positive fluid thrill without tenderness. The skin was taut with pitting edema extending to the mid-shin, but no petechiae, rashes, or lesions were noted.

Laboratory and imaging findings

**Figure 1:**
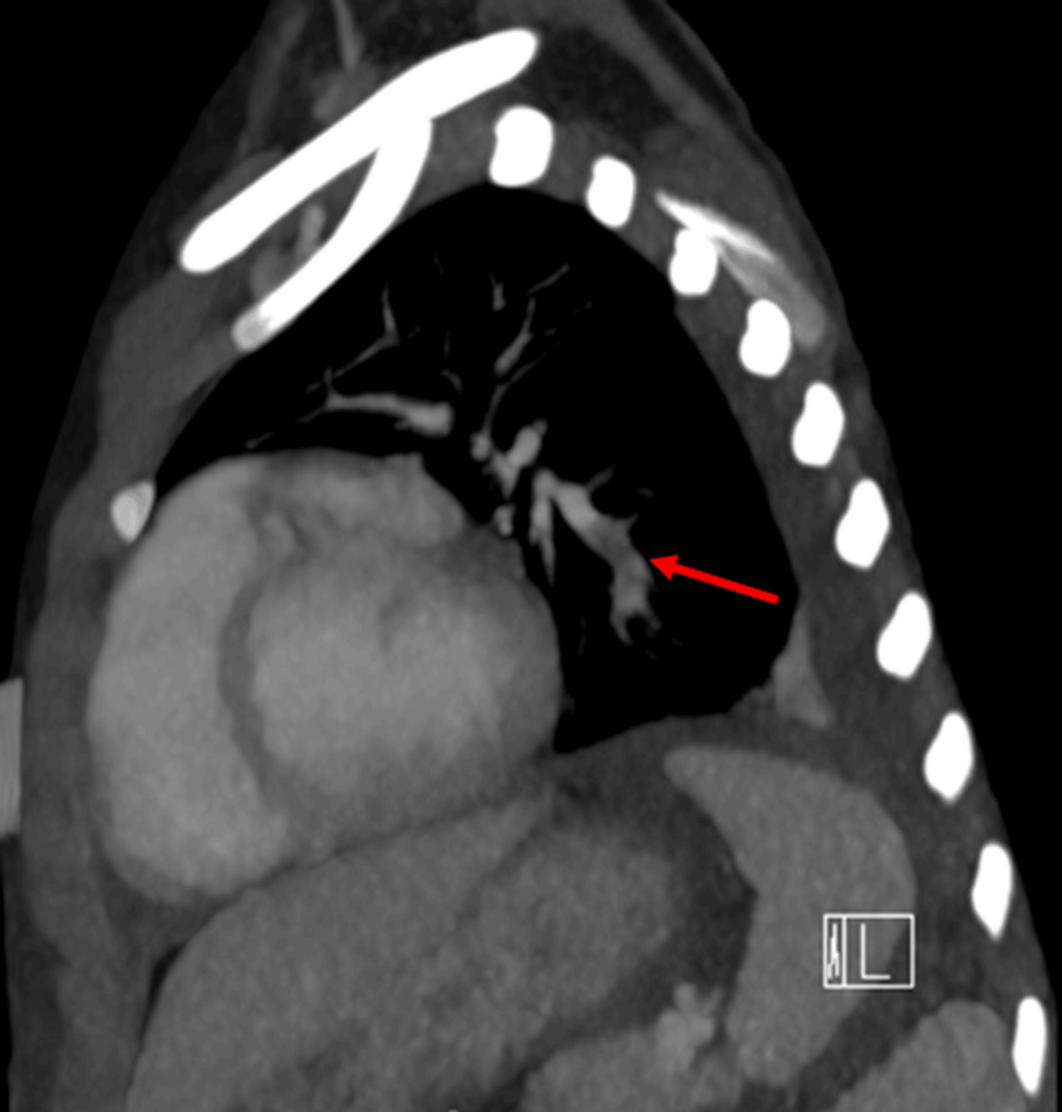
Acute occlusive pulmonary thromboembolism involving left basilar lateral and posterior segmental branches in sagittal view indicated by red arrow.

**Figure 2:**
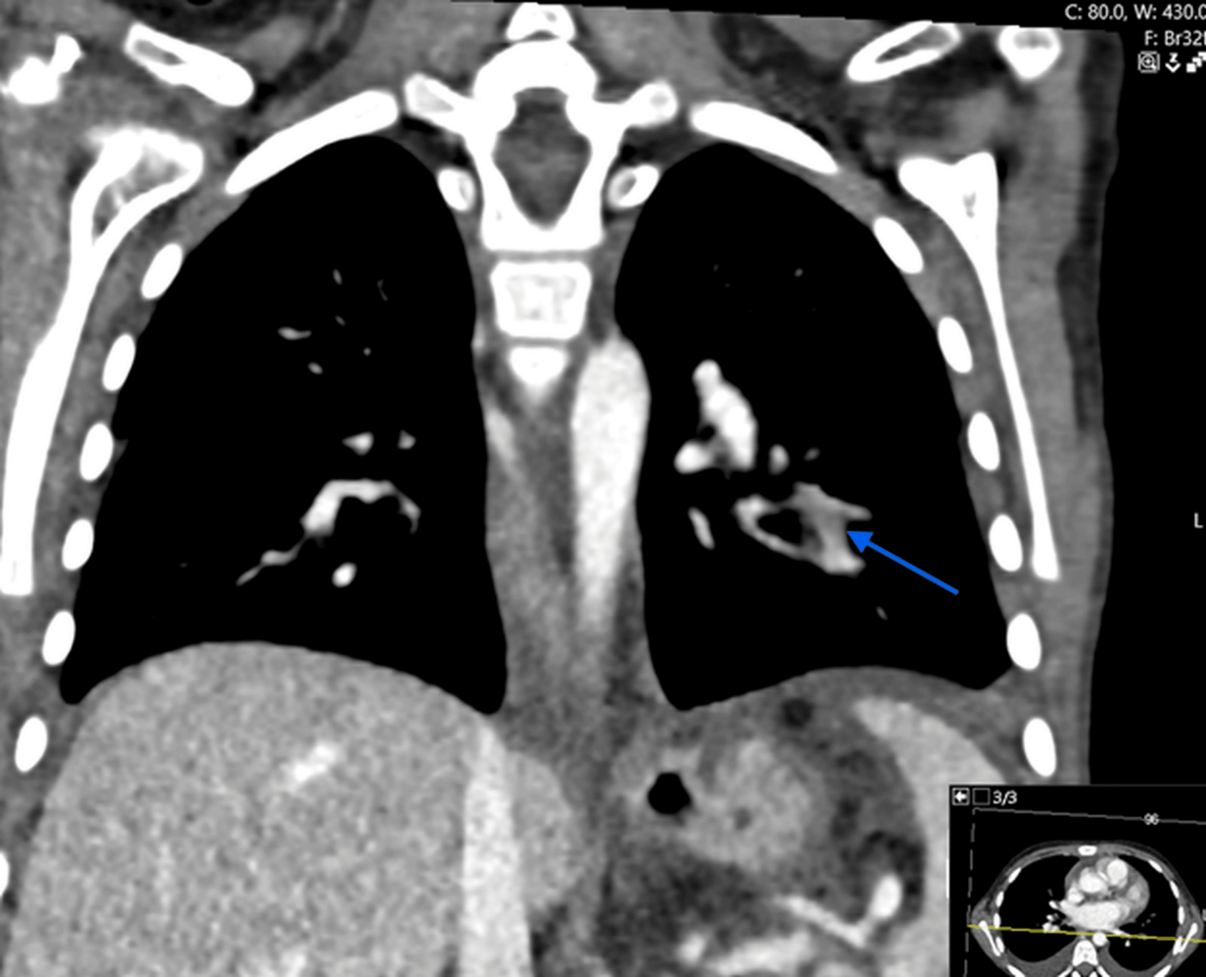
Acute occlusive pulmonary thromboembolism involving left basilar lateral and posterior segmental branches in coronal view indicated by blue arrow.

Given her symptoms of chest pain/pressure, palpitations, and dyspnea with underlying SRNS, PE was suspected. A CT angiogram of the lungs was performed and demonstrated an occlusive pulmonary thromboembolus (PTE) involving the left basilar lateral and posterior segmental branches, as shown in Figures 1-2. Echocardiogram demonstrated a small ASD/PFO with left-to-right shunt, mild mitral regurgitation, mild to moderate aortic insufficiency, and no evidence of right ventricular strain. Upon detection of pulmonary VTE on CT angiogram, a venous Doppler US of the lower extremities was conducted to rule out deep vein thrombosis (DVT), which was negative. Initial laboratory evaluation included cardiac markers, coagulation studies, complete blood count, comprehensive metabolic panel, and urine studies, including a 24-hour urine protein collection. Pertinent results are summarized in Table 1.

**Table 1:** Significant lab results compared to normal reference range from investigations in our patient.

Investigation	Patient’s value	Reference range
Troponin	0.077 ng/mL	0-0.034 ng/mL
N-terminal pro-B-type natriuretic peptide	6,930 pg/mL	0-300 pg/mL
Activated partial thromboplastin time (APTT)	51.8 seconds	23.3-38.6 seconds
D-dimer (fibrin degradation product)	8.91 µg/mL	0-0.50 µg/mL
White blood cells	33,000 /µL	4,500-11,000 /µL
Platelet count	504,000 /µL	150,000-450,000 /µL
Serum sodium	129 mmol/L	135-145 mmol/L
Serum albumin	1.6 g/dL	3.5-5 g/dL
Serum creatinine	0.5 mg/dL	0.2-0.8 mg/dL
Corrected serum calcium	8.7 mg/dL	8.4-10.2 mg/dL
Serum blood urea nitrogen	33 mg/dL	7-17 mg/dL
Urine protein	>300 mg/dL	None
Urine blood	Large	None
Urine RBCs	5-10 /hpf	None
24-hour urine protein	5,412 mg/day	42-225 mg/day

Hospital course and treatment

Following confirmation of PE, the patient was admitted to the pediatric intensive care unit (PICU). She remained hemodynamically stable and did not require supplemental oxygen. Anticoagulation was initiated with intravenous unfractionated heparin (UFH) (loading dose 75 units/kg, maintenance 20 units/kg/hr), followed by transition to enoxaparin (low-molecular-weight heparin, or LMWH) 1 mg/kg twice daily once stable. Anti-Xa levels were monitored to maintain the therapeutic range of 0.5-1 anti-Xa units/mL.

Given her significant edema, she received furosemide twice daily and 25% albumin, followed by furosemide on two occasions for diuresis. Blood pressure was controlled without the need for continuous antihypertensive infusions.

Renal biopsy results confirmed primary focal segmental glomerulosclerosis (FSGS) with a collapsing glomerular lesion. Given the patient’s poor response to high-dose corticosteroids and tacrolimus, the diagnosis of collapsing FSGS, a high-risk histologic subtype, and the development of a thromboembolic complication in the setting of persistent NS, escalation of therapy was warranted. Therefore, rituximab was initiated during this admission at a dose of 375 mg/m² weekly for four doses, targeting refractory disease and ongoing severe proteinuria. Renasight gene panel was negative, but carrier NPHS2 congenital nephrotic syndrome, type 2, autosomal recessive, c.988_989del (p.Leu330Valfs*15), heterozygous, likely pathogenic.

## Discussion

In our case, we present a patient with SRNS who developed a PE after recently being hospitalized and undergoing a kidney biopsy. The kidney biopsy, as well as genetic testing for our patient, were performed as advised in KDIGO guidelines for the management of children with SRNS to help guide appropriate management of the underlying disease [7,8]. Our patient was found to have SRNS secondary to FSGS, a condition associated with high morbidity and a propensity for chronic renal impairment.

As evidenced in this case, children with NS are at increased risk of developing a life-threatening TE due to developing a hypercoagulable state through severe proteinuria in which anticoagulant proteins are lost in urine [1,2,5]. In our patient, through extended lab work performed after detection of her PE, revealed the following hematologic profile: elevated protein C at 119%, Factor V Leiden mutation negative, prothrombin gene mutation negative, antithrombin III antigen low 72, protein S antigen free low 32%, protein S antigen total normal 136%.

SRNS is associated with a higher incidence of TE compared to steroid-sensitive variants [1]. Thus, it is crucial to recognize additional risk factors that further increase this risk, several of which were present in our patient, including steroid use; intravascular volume depletion, evidenced by tachycardia, tachypnea, and pitting edema; prolonged immobility due to being confined to a hospital bed for most of her hospital stay; and diuretic use [1,2,5]. Furthermore, infections significantly increase thrombosis risk in SRNS due to the urinary loss of circulating antibodies [9].

Recent epidemiological studies highlight the importance of early recognition and management of TE in patients, particularly older children and adolescents with significant proteinuria, to prevent fatal outcomes [1]. Identification of the signs and symptoms associated with PTE, its diagnosis via CT angiography, which was ordered promptly for our patient presenting with cardiopulmonary symptoms consistent with a PE, are crucial to reducing morbidity and mortality associated with TE events in children with NS [10]. Further imaging, including a venous doppler US to check for DVTs and an echocardiogram to evaluate for right heart strain, neither of which was present in our patient, was conducted during the acute stage to guide management decisions and assess the severity of the patient’s condition. Circulatory failure, right ventricular dysfunction, and hemodynamic instability can occur in cases of massive PTE [11].

A high plasma D-dimer level in children with NS is associated with PTE formation, but in high-risk patients, it is not specific but is more helpful in ruling out VTE [12]. Moreover, D-dimer levels in patients with NS are routinely elevated at baseline, and rather than an indication of thrombosis, levels are increased proportionally to the severity of proteinuria and inversely proportional to hypoalbuminemia [13].

According to the American College of Chest Physicians (ACCP) guidelines on antithrombotic therapy in neonates and children, prompt initiation of fast-acting anticoagulation with UFH is recommended, which was quickly initiated in our patient after detection of PE on CT angiography, followed by continued anticoagulation with LMWH for at least three to six months or until the precipitating risk factor is resolved [1,14]. Furthermore, the role of thrombolytics in children remains limited, with their use only recommended in limb- or life-threatening VTE [14]. There is insufficient data to support prophylactic anticoagulation in most cases of pediatric NS, despite the life-threatening complication of thrombosis, and it is a grade C, weak recommendation according to the IPNA clinical practice recommendations and ungraded according to KDIGO guidelines [6-8]. However, in SRNS patients with a history of TE prophylactic anticoagulation with LMWH should be continued as above, and if required for a prolonged period, can be bridged to a vitamin K antagonist [2,8].

Therefore, mitigating risk factors associated with thrombosis should be prioritized in patients with NS whenever possible. Encouraging mobility during hospitalization and emphasizing the importance to patients and families, and when patients must be immobile for long periods of time, to use sequential compression devices and work closely with physical therapists to optimize mobility wherever possible. Central venous catheter use is a known significant risk factor for TE in children and should be avoided if possible and, if required, removed once no longer medically necessary. Similarly, diuretic use should be administered diligently with direct parameters such as strict fluid balance goals and be avoided in patients with evidence of intravascular volume depletion, such as tachycardia, oliguria, prolonged capillary refill, and hypotension due to increased thrombosis risk [8]. Balancing the administration of glucocorticoids in SRNS patients who have severe proteinuria and, as previously stated, puts them at increased risk of TE, which requires aggressive treatment to go into remission with the risk of TE as an adverse effect of glucocorticoids themselves through steroid-sparing agents as outlined in KDIGO guidelines [8]. In addition, given the high risk of infection and its close association with TE, prompt initiation of empiric antibiotics covering *Streptococcus pneumoniae* is recommended in hospitalized NS children only with high suspicion of infection [9]. Finally, when risk factors cannot be mitigated, high vigilance for the development of TE is crucial.

## Conclusions

Prompt recognition and treatment of PE in our patient led to a successful outcome, underscoring the importance of early diagnosis, rapid imaging, and timely anticoagulation. Pediatricians should remain vigilant for TE in children with NS and address modifiable risk factors, especially when hospitalized, to reduce the incidence and continue anticoagulation in patients with a history of PE as indicated on an individual basis with collaboration with a hematologist to prevent the recurrence while balancing bleeding risk. Educating families on the symptoms of PE and DVT is crucial for timely intervention. Further research is needed to guide routine imaging, identify high-risk patients, evaluate prophylactic anticoagulation, and improve treatment strategies to reduce TE risk in SRNS.
